# *Medicago truncatula *and *Glomus intraradices *gene expression in cortical cells harboring arbuscules in the arbuscular mycorrhizal symbiosis

**DOI:** 10.1186/1471-2229-9-10

**Published:** 2009-01-22

**Authors:** S Karen Gomez, Hélène Javot, Prasit Deewatthanawong, Ivone Torres-Jerez, Yuhong Tang, Elison B Blancaflor, Michael K Udvardi, Maria J Harrison

**Affiliations:** 1 Boyce Thompson Institute for Plant Research, Tower Road, Ithaca, NY 14853, USA; 2 Plant Biology Division, Samuel Roberts Noble Foundation, 2510 Sam Noble Parkway, Ardmore, OK 73401, USA; 3 CEA/Cadarache IBEB, Service de Biologie Végétale et de Microbiologie Environnementales, UMR 6191 CNRS-CEA-Aix Marseille Univ., F-13108 St. Paul Lez Durance, France

## Abstract

**Background:**

Most vascular flowering plants have the capacity to form symbiotic associations with arbuscular mycorrhizal (AM) fungi. The symbiosis develops in the roots where AM fungi colonize the root cortex and form arbuscules within the cortical cells. Arbuscules are enveloped in a novel plant membrane and their establishment requires the coordinated cellular activities of both symbiotic partners. The arbuscule-cortical cell interface is the primary functional interface of the symbiosis and is of central importance in nutrient exchange. To determine the molecular events the underlie arbuscule development and function, it is first necessary to identify genes that may play a role in this process. Toward this goal we used the Affymetrix GeneChip^® ^Medicago Genome Array to document the *M. truncatula *transcript profiles associated with AM symbiosis, and then developed laser microdissection (LM) of *M. truncatula *root cortical cells to enable analyses of gene expression in individual cell types by RT-PCR.

**Results:**

This approach led to the identification of novel *M. truncatula *and *G. intraradices *genes expressed in colonized cortical cells and in arbuscules. Within the arbuscule, expression of genes associated with the urea cycle, amino acid biosynthesis and cellular autophagy was detected. Analysis of gene expression in the colonized cortical cell revealed up-regulation of a lysine motif (LysM)-receptor like kinase, members of the GRAS transcription factor family and a symbiosis-specific ammonium transporter that is a likely candidate for mediating ammonium transport in the AM symbiosis.

**Conclusion:**

Transcript profiling using the Affymetrix GeneChip^® ^Medicago Genome Array provided new insights into gene expression in *M. truncatula *roots during AM symbiosis and revealed the existence of several *G. intraradices *genes on the *M. truncatula *GeneChip^®^. A laser microdissection protocol that incorporates low-melting temperature Steedman's wax, was developed to enable laser microdissection of *M. truncatula *root cortical cells. LM coupled with RT-PCR provided spatial gene expression information for both symbionts and expanded current information available for gene expression in cortical cells containing arbuscules.

## Background

The arbuscular mycorrhiza is the name given to a symbiotic association of plants and arbuscular mycorrhizal (AM) fungi. The AM symbiosis occurs widely throughout the plant kingdom and involves angiosperms, gymnosperms, pteridophytes and some bryophytes. The fungi that participate in this symbiosis are all members of the Glomeromycota, [1-3], a likely sister group to the Ascomycota and Basidiomycota. The symbiosis develops in the plant roots, where AM fungi form extensively branched hyphae, called arbuscules, in the cortical cells. In addition to growth within the root, the fungus develops a network of extra-radical hyphae that extends into the rhizosphere. Inorganic phosphate (Pi) and nitrogen (N), acquired by the extra-radical hyphae, are translocated to the arbuscules and released to the plant. In return the plant provides the fungus with carbon. P and N are the two mineral nutrients that plants require in the greatest quantities and therefore the symbiosis has an important influence on plant health and consequently on ecosystem function [4-6].

There has been significant progress in understanding the signaling associated with the early stages of development of AM symbiosis [7]. Some of the signal molecules have been identified, including strigolactones, which act as pre-contact signals to stimulate AM fungal growth and metabolism [8,9]. In addition, several components of a plant 'symbiosis signaling' pathway required for fungal entry into the root have been cloned [10-17]. These genes were identified initially in legumes, where the symbiosis signaling pathway is required also for the interaction with rhizobia. However, orthologs of these genes have been identified in tomato and rice, where they are required for AM symbiosis [18,19]. This further supports the hypothesis that the symbiosis signaling pathway arose initially for interactions with AM fungi, and in legumes it was co-opted to enable interactions with rhizobia [19-21]. Currently, for AM symbiosis, the downstream genes controlled by this pathway are not known. In general, events associated with fungal development in the cortex are less well understood, and genes controlling arbuscule formation, have yet to be identified. Arbuscule development is an active process for both symbionts. The fungal hypha penetrates the cortical cell wall and undergoes repeated dichotomous branching, while the plant cell undergoes significant cellular changes, including reorganization of the cytoskeleton, endoplasmic reticulum and development of the peri-arbuscular membrane, which envelops the arbuscule [22-26]. Reorganization of the cytoskeleton and endoplasmic reticulum is initiated prior to arbuscule formation suggesting that a mobile signal may trigger these events [26,27]. Currently, information about the lipid and protein content of the peri-arbuscular membrane is limited, but it is clear that the membrane contains some unique proteins, including symbiosis-specific Pi transporters that function to import Pi into the cortical cell [28]. The transporters involved in N acquisition are as yet unknown, but a series of nuclear magnetic resonance (NMR) spectroscopy and gene expression studies indicate that the fungus assimilates N in the external hyphae, translocates this to the intra-radical hyphae as arginine, and then disassembles the arginine to retain the carbon molecules, while providing N to the plant, probably as ammonium (NH_4_^+^) [29-31]. Although not shown directly, it is likely that NH_4_^+ ^transfer to the plant occurs at the arbuscule-cortical cell interface also.

Arbuscules are terminally differentiated hyphae with a finite life and following development, they undergo a degeneration phase in which the arbuscule branches collapse, and the arbuscule gradually disappears from the cell [22,32-34]. This process is not well understood, but it is clear that arbuscule development and degeneration occur cell autonomously and arbuscules in neighboring cells may be in different phases of the life cycle. Although the arbuscule-cortical cell interface is central to the AM symbiosis, currently, we understand little of the processes that enable its development or that control its degeneration.

To gain insight into the molecular changes that accompany development of AM symbiosis, a variety of transcript profiling approaches have been undertaken. In rice, surveys using a whole genome chip identified mycorrhiza-responsive genes and revealed a partial overlap in the transcriptional responses to mutualistic and pathogenic biotrophic fungi [35]. In *Medicago truncatula*, suppressive subtractive hybridization, cDNA arrays and long oligonucleotide arrays have been used to document transcript changes associated with AM symbiosis [36-40]. The inclusion of *M. truncatula *symbiosis mutants enabled gene expression to be associated with different phases of the symbiosis, for example appressoria formation [41]. A mycorrhizal root includes a heterogeneous mix of colonized and non-colonized cells, and therefore spatial gene expression information is particularly important. *In situ *hybridization and/or analysis of promoter-reporter fusions demonstrated that genes whose expression was induced in colonized roots could be further separated into two groups; those expressed exclusively in cells containing arbuscules and those expressed in cells containing arbuscules and in neighboring non-colonized cells [28,36,38,42-46]. These data indicate that the different cortical cells of the mycorrhizal root system have unique transcript profiles, and point to at least two signaling pathways active in regulating mycorrhiza-induced gene expression. While these approaches have advanced information about gene expression in colonized cortical cells, *in situ *hybridization or promoter-reporter gene studies are labor intensive and this precludes surveys of a large number of genes. Our current knowledge of genes expressed in cells containing arbuscules is limited to less than 35 genes. In addition, the genes that have been analyzed are those that have the highest transcript levels and relatively few receptors or transcriptional regulators have been investigated. Beyond plant gene expression in the colonized cortical cell, almost nothing is known about gene expression in the arbuscule.

Laser microdissection (LM) offers a new way to obtain RNA from a subset of cells, and coupled with transcript profiling approaches, it provides an opportunity to monitor gene expression in an individual cell type more easily than promoter-reporter gene fusions. LM was originally developed to isolate specific animal cells from complex tissues; a breakthrough for cancer and neuronal research [47,48]. In plants, LM was used initially to isolate phloem cells from rice leaves [49]. Subsequently, epidermal and vascular tissues were isolated from maize coleoptiles [50] and then various cell or tissue types, including bundle sheath, shoot tip protoderm, shoot apical meristem, leaf primordium, seedling procambium, hypocotyls and root meristems have been harvested from maize, rice, radish and tomato [51,52]. So far, most studies have focused on shoot tissues. In a few cases, LM has been used to isolate plant cells during interactions with other organisms including cyst or root-knot nematode feeding sites in soybean roots [53-57]. In addition, fungus-plant interactions were assessed in maize by dissecting parenchyma cells infected with *Colletotrichum graminicola *and the RNA samples were used for analysis of *C. graminicola *gene expression [58]. Recently, this technology was used to isolate cells from arbuscular mycorrhizal or non-mycorrhizal tomato roots [59] where it enabled the analysis of phosphate transporter gene expression in the plant and the fungus.

Toward the ultimate goal of understanding how arbuscules develop, we initiated experiments to identify genes expressed in cortical cells containing arbuscules. We first undertook a profiling experiment with a new *M. truncatula *GeneChip^®^, and then developed LM of *M. truncatula *root cortical cells to enable cell-type specific analyses of gene expression by RT-PCR. This approach led to the identification of novel plant and fungal genes expressed in colonized cortical cells and in arbuscules, including members of the LysM-receptor like kinase family, GRAS transcription factor family, a novel ammonium transporter that is a strong candidate for mediating symbiotic ammonium transport, and AM fungal genes of the urea cycle.

## Results

### Use of the Medicago GeneChip^® ^to monitor transcript profiles in *M. truncatula *colonized by *Glomus intraradices*

Previously in *M. truncatula*, a long oligonucleotide-based array containing 16,000 features, was used to profile gene expression in a number of different AM symbioses [38,39]. While these studies provided important insights into gene expression during the AM symbiosis, they surveyed only a small fraction of the *M. truncatula *transcriptome. In order to expand these gene expression studies, we used the Affymetrix Medicago GeneChip^®^. It contains 61,278 probe sets of which 32,167 were designed based on sequences from the *M. truncatula *EST database and 18,733 were based on gene predictions from the International Medicago Genome Annotation Group (IMGAG) . The GeneChip^® ^was hybridized with three biological replicate RNA samples from *M. truncatula *and *M. truncatula*/*G. intraradices *mycorrhizal roots and using significance criteria established previously [60], 652 genes that showed a 2-fold or greater change in gene expression in mycorrhizal roots relative to the mock-inoculated controls were identified. Of these, 637 genes showed increased transcript levels in *G. intraradices*-colonized roots, and 15 genes showed decreased transcript levels (Additional file 1). Our previous experiments with the *M. truncatula *16K array had identified 110 genes that showed a 2-fold or greater change in gene expression [39]. 82 induced genes were present in both data sets [39]. Thus, the GeneChip dataset has extended the transcriptional information available for *M. truncatula/G. intraradices *mycorrhizal roots substantially.

New AM-induced genes with potentially important roles in the symbiosis include an ammonium transporter (IMGAG|1723.m00046), which shows a 4.8 fold induction in mycorrhizal roots (Table 1). This transporter is a candidate for mediating symbiotic N transport. Other transporters including a zinc transporter (AJ499751, 7.6 fold), organic-cation transporter (TC98622, 2.5 fold), and copper transport protein (TC97522, 50 fold) were induced in mycorrhizal roots, along with genes encoding transporters of the peptide transporter family (AL377202, TC97569, AL387494, and BE998753) and ABC transporter family (AL382570, IMGAG|1050.m00013, IMGAG|733.m00003, IMGAG|1088.m00025, AW287942, BG644663, TC109985, TC96634, IMGAG|795.m00013).

**Table 1:** Gene expression data for genes selected for analysis in the LM cell samples.

		**Array data**		
		
	**Annotation**	**Ratio^1 ^(Mt/Gi-Mt)**	**P-Value**	**q-Value**
***M. truncatula *Genes**				
TC100851	*MtNIP1*	15.71	0	0.030
TC101391	Bark lectin II precursor^a^	72.70	0	0.084
TC107070	Blue copper-binding protein-like^a^	162.37	0	0.164
TC98064	Cysteine-rich antifungal protein 2 precursor	47.05	0	0.030
TC101060	Defensin AMP1	130.10	0	0.029
TC106954	*MtSCP1*	29.94	0	0.044
TC101627	Transfactor-like protein	195.78	0	0.030
TC94453	*MtPT4*	158.20	0	0.030
AJ499899	GRAS family member	99.58	0	0.054
TC97569	Peptide transporter	7.21	0	0.028
TC108660	GDSL-motif lipase/hydrolase-like protein	9.24	0	0.038
TC99271	Palmitoyl-acyl carrier protein thioesterase	49.41	0	0.064
1197.m00015	*MtLYR1*	3.65	0	0.091
AL386880	GRAS family member	7.41	1.6E-171	0.072
TC110731	Triacylglycerol/steryl ester lipase-like protein	102.45	0	0.157
TC97522	Copper transport protein	50.49	0	0.035
TC104979	Dihydrodipicolinate synthase	15.55	0	0.030
906.m00010	Inositol polyphosphate related phosphatase	4.46	0	0.112
AL387494	Peptide transporter 2	6.73	0	0.043
AW573798	SNF1-related protein kinase	7.72	0	0.140
1723.m00046	Ammonium transporter	4.78	4.4E-154	0.110
				
**Putative *G. intraradices *Genes**				
TC112506	ADP-ribosylation factor	25.84	0	0.036
TC105524	Probable autophagy protein AUT7	11.06	0	0.031
TC111688	Related to p24 protein	38.10	0	0.029
AL389511	Glutamine synthetase^a^	118.80	0	0.043
TC105031	Arginase	2.43	8.33E-39	0.135
TC109807	Probable ornithine aminotransferase	4.20	1.6E-186	0.111
AL387743	Argininosuccinate synthase	10.45	0	0.058
TC100037	Asparagine synthetase	19.56	0	0.100
TC106186	Probable acyl-CoA dehydrogenase	10.66	0	0.031
AL382096	Potential phospholipid-transporting ATPase DRS2	5.25	0	0.182
TC105246	Delta-9 fatty acid desaturase	140.11	0	0.030

^a^Genes tested by RT-PCR have different ID

^1^Expression ratio for genes from the Medicago GeneChip^® ^experiment that showed differential expression in *M. truncatula *roots colonized by *G. intraradices *relative to mock-inoculated control roots. For complete data set see Additional file 1.

Carbon metabolism and carbon transport in the AM symbiosis are also areas of great importance that are currently poorly understood. A putative alpha-amylase gene (IMGAG|1739.m00052) was up-regulated 2.2 fold suggesting a potential increase in starch breakdown. Also, a putative SNF1-related protein kinase gene (AW573798) was up-regulated 7.7 fold (Table 1). The SNF1 and SNF1-related kinases are widely conserved in eukaryotes and play a central role in sugar and energy signaling [61].

Two genes, one predicted to encode a protein involved in lipid biosynthesis, palmitoyl-ACP thioesterase, and one encoding an enzyme of lipid breakdown, a triacylglycerol/steryl ester lipase (TC110731) were highly up-regulated. The latter showed a 102-fold increase in transcript levels (Table 1). This increase in both biosynthetic and degradative activities may be associated with the ongoing development and breakdown of the peri-arbuscular membrane associated with the different phases of the arbuscule lifespan.

Currently, transcription factors that regulate AM-induced gene expression are unknown. Here, 5 probesets representing members of the GRAS family of transcription factors (AL386879, AL386880, TC105118, IMGAG|1755.m00026 and AJ499899) indicate that several members of this gene family are up-regulated more than 7-fold. Two GRAS family transcription factors are essential for nodulation [62,63], and given the conserved symbiosis signaling pathway, there is a strong possibility that members of this family will be involved in regulating gene expression in AM symbiosis also. Other genes related to signaling or perception of signal, include a putative inositol polyphosphate phosphatase (IMGAG|906.m00010) that was up-regulated 4.4-fold (Table 1). Inositol polyphosphate 5-phosphatases are involved in the modulation of phosphoinositide signaling. In plants, inositol polyphosphate 5-phosphatases are members of large gene families and their individual roles are mostly unknown. While this signaling pathway has not been implicated in the AM symbiosis, lysophosphatidyl choline, was recently shown to induce expression of symbiotic Pi transporters in potato and tomato [64]. Finally, one of the most intriguing findings was a gene encoding a lysine motif-receptor-like kinase (LysM-RLK) called *MtLYR1 *[65] which showed a 3.6-fold increase in mycorrhizal roots (Table 1). *MtLYR1 *is a close relative of *NFP*, a LysM-RLK gene that is likely a Nod factor receptor [65,66].

### *Glomus intraradices *genes are represented on the Medicago GeneChip^®^

**Figure 1:**
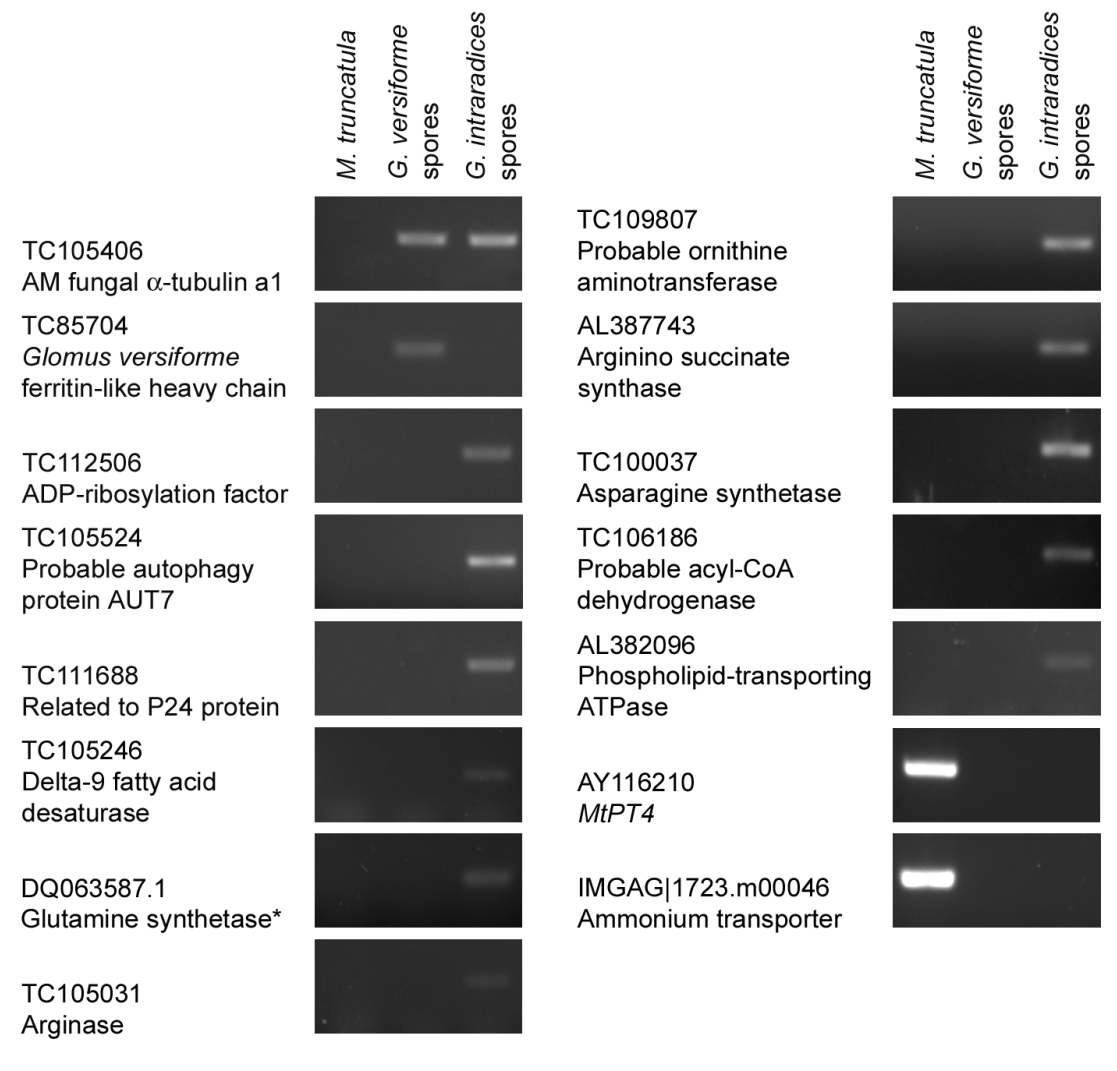
**Analysis of the origin of putative *G. intraradices *genes represented on the *Medicago *GeneChip^®^**. PCR amplification using DNA isolated from *M. truncatula *roots, *G. versiforme *spores or *G. intraradices *spores. *MtPT4 *and an ammonium transporter are positive controls for the *M. truncatula*. Primers designed to TC105406, a putative α-tubulin a1 gene amplify α-tubulin from both *G. intraradices *and *G. versiforme*. TC85704 is a *G. versiforme *gene.

The Medicago GeneChip^® ^includes 32,167 probes designed to sequences from the *M. truncatula *Gene Index . The Gene Index includes ESTs from mycorrhizal root cDNA libraries, consequently, there is the potential that some probes were designed, unintentionally, to ESTs that represent genes from the AM fungal symbionts. The discovery that some probes represent genes with BLAST hits exclusively to fungal sequences suggested that this may be the case. Therefore, we systematically mined the array data to identify probes that might represent AM fungal genes. Probes that showed an average expression value on the mock-inoculated arrays near background levels (≤ 63) and a higher average expression value on *G. intraradices*-inoculated arrays, and corresponded to ESTs present exclusively in mycorrhizal cDNA libraries, were considered strong candidates. Subsequent TBLASTX analysis indicated that 49 probesets with the criteria outlined above represented genes that showed best BLAST hits to fungal sequences (Additional file 2). There were additional probes designed to ESTs that showed similarity to fungal sequences but the ESTs were short and the BLAST scores were unreliable, so they were excluded from further analysis. Of the 49 genes identified as putative fungal genes, 22 are represented by a single EST, and all are present exclusively in *M. truncatula/G. intraradices *mycorrhizal root cDNA libraries and are therefore likely to represent *G. intraradices *genes. Experimental analysis of ten genes confirmed that they are *G. intraradices *genes. They could be amplified by PCR from *G. intraradices *genomic DNA and not from *M. truncatula *genomic DNA (Table 1; Figure 1). Except for the putative *G. intraradices *a-tubulin a1 gene, which is highly conserved, the primers designed to the *G. intraradices *genes did not amplify transcripts from *G. versiforme*. It has been shown that despite a common genus name, these two fungi are only distantly related [67]. Eight of these genes (TC100037, TC103687, TC104499, TC105031, TC109807, TC111242, TC112506 and TC105246) were represented on the 16K array although their origin was not demonstrated [39].

Previous estimates suggest that total RNA extracted from a highly colonized, whole root system contains not more than 12% AM fungal RNA [68]. Consequently, it might be predicted that the fungal transcripts represented in the EST libraries and consequently represented on the GeneChip, are likely to represent the most highly expressed fungal genes. Interestingly, the fungal genes represented on the array include genes predicted to encode enzymes of N metabolism, specifically, the urea cycle (Additional file 2).

### Development of a laser microdissection procedure suitable for the capture of cortical cells from *M. truncatula *mycorrhizal roots

To enable LM of cortical cells containing arbuscules, the first issue was to identify suitable regions of the root system to fix and embed. In an AM symbiosis, the fungus proliferates in the inner cortex of the root and in *M. truncatula *mycorrhizal roots, the colonized regions are not readily visible without fixing and staining. In particular, it is difficult to identify regions of the root with infections that contain large numbers of arbuscules. To facilitate this, our goal was to first develop a *M. truncatula *transgenic plant line in which the colonized regions of the root would be marked by expression of green fluorescent protein (GFP). Previously, we had identified *MtSCP1*, a gene that is expressed only in mycorrhizal roots, specifically in colonized regions of the roots [36]. Analysis of an *MtSCP1 *promoter-GFP fusion construct in *M. truncatula *transgenic roots indicated that the construct reported the presence of the AM fungus in the cortex [36]. The *MtSCP1 *promoter-GFP fusion construct was transferred to *Agrobacterium tumefaciens *and *M. truncatula *transgenic plants were generated. The transgenic plants showed the same expression patterns as seen previously and strong GFP fluorescence reported the presence of the fungus in the cortex (Additional file 3). Using the transgenic plants, we were able to identify and dissect out highly colonized regions of the root. These were then used for LM experiments. In addition, RNA extracted from these intact colonized root pieces is highly enriched for plant and fungal transcripts associated with colonized regions of the root, and are useful RNA resource.

**Figure 2:**
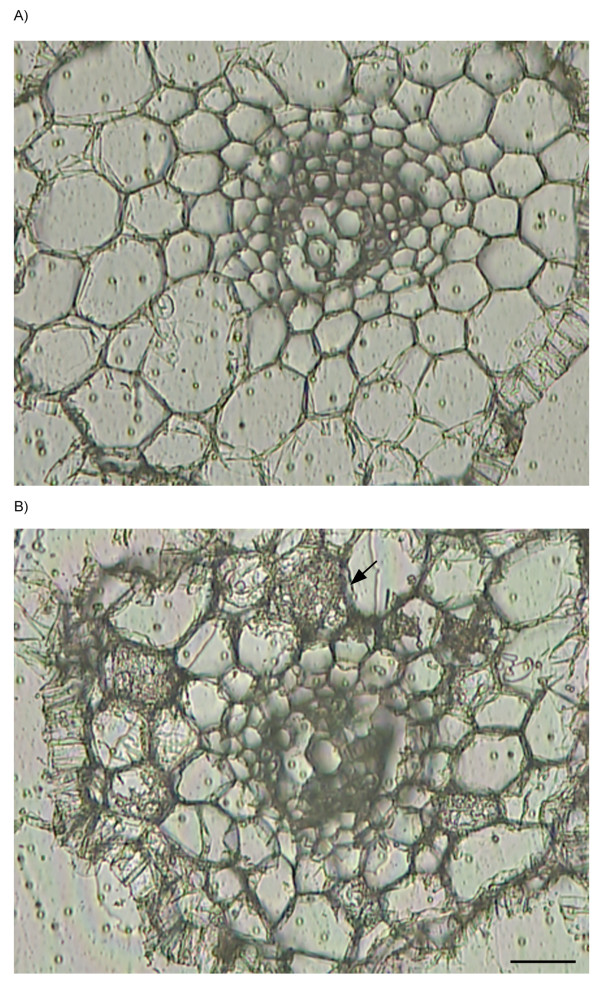
**Transverse sections of *M. truncatula *roots used for laser microdissection**. Mock-inoculated controls (A) and inoculated with *G. versiforme *(B). The arrow shows a cortical cell containing an arbuscule. Bar = 25 μm

A review of LM and its use in plants, revealed that a wide variety of protocols have been used for the preparation of plant tissue for LM [52]. Each different plant tissue has unique characteristics and therefore it is necessary to determine empirically, the most appropriate fixation and embedding regime to retain satisfactory cellular morphology, while still enabling extraction of high RNA quality. We based our approach on a protocol developed by Kerk et al. [51] and then evaluated aspects of fixation, embedding, RNA extraction and amplification to find conditions optimal for *M. truncatula *mycorrhizal roots. RNA quality was evaluated using a bioanalyzer and/or agarose gel electrophoresis. Tissues were fixed in Farmer's fixative and fixation times of 12 or 24 h yielded RNA with an RNA integrity number (RIN) of 8.5 (intact RNA, RIN = 10) as assessed by a bioanalyzer. In addition, fixation at 12 and 24 hours yielded larger size amplified, antisense RNA (aRNA) molecules, than a 4 h fixation (data not shown). We experienced considerable difficulties with RNA degradation during the paraffin embedding and xylene de-waxing stages, so as an alternative to paraffin wax, we tested Steedman's wax, a polyester wax used for immunolocalization [69-71]. Steedman's wax has the advantages that it can be mixed under RNase-free conditions, has a lower melting temperature (38–40°C), and sections can be de-waxed easily with ethanol. The RNA quality extracted from root samples embedded in Steedman's was good (RIN = 6.7) and the morphology of the sections were comparable to sections made from tissues embedded in paraffin wax (Figure 2).

**Figure 3:**
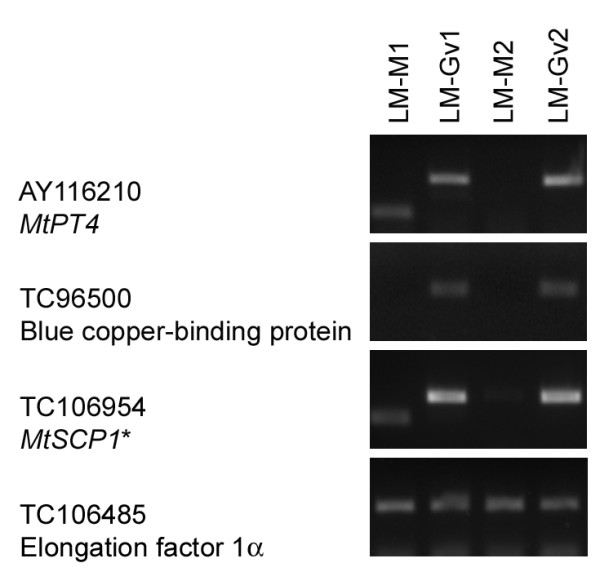
**RT-PCR analysis to detect transcripts of *MtPT4*, *MtBCP1 *and *MtSCP1 *in colonized cortical cells**. RNA from cortical cells from two independent mock-inoculated *M. truncatula *root systems (LM-M1 and LM-M2) and cortical cells containing arbuscules from *M. truncatula*/*G. versiforme *mycorrhizal root systems (LM-Gv1 and LM-Gv2) was used. Cortical cells were obtained by laser microdissection (LM). *Two rounds of PCR were carried out for TC106954. A *M. truncatula *elongation factor 1α (TC106485) was included as endogenous control.

LM and laser pressure catapulting was carried out with the P.A.L.M. microbeam system. Between 400 and 600 cortical cells were harvested directly into lysis buffer and RNA was isolated within 12 hours of harvest. Two rounds of linear RNA amplification yielded over 23 μg of aRNA (Additional file 4). To validate the LM RNA, we examined the expression of *MtPT4*, a gene expressed exclusively in cells containing arbuscules. In addition, we assessed expression of *MtSCP1*, and *MtBCP1*, two genes expressed in this cell type that are suitable as additional markers. The latter two genes are not unique to colonized cortical cells [28,36,38]. *MtPT4*, *MtSCP1*, and *MtBCP1 *transcripts were detected in two biological replicate samples of RNA from cells with arbuscules and not from cortical cells captured from mock-inoculated roots. Elongation factor 1-alpha (*EF1α*) transcripts were present in RNA samples from both cell types (Figure 3). The length of individual molecules present in the aRNA sample was evaluated by examining *MtPT4 *transcripts [28]. cDNA was synthesized using random priming and the length of molecules in the samples was evaluated by RT-PCR using primers distributed across the *MtPT4 *gene. Although we were unable to amplify the intact 1.8 kb *MtPT4 *transcript, primers sets that amplified less than 210 bp fragments at the 5' end, middle, or 3' end of the coding sequence showed that the aRNA represents complete transcripts but not necessarily as single intact molecules (Additional file 5).

### *M. truncatula *gene expression in cortical cells containing *G. versiforme *and *G. intraradices *arbuscules

**Figure 4:**
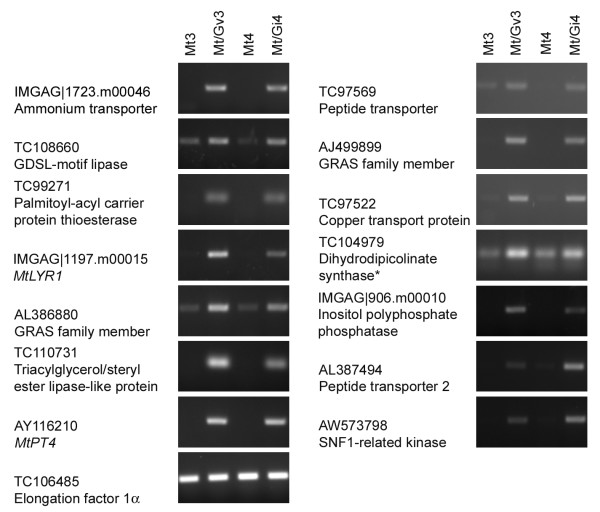
**RT-PCR analysis to detect transcripts of thirteen *M. truncatula *genes using RNA from mock-inoculated roots, and from mycorrhizal roots**. RNA from root pieces from *M. truncatula *mock-inoculated roots (Mt3) or *M. truncatula*/*G. versiforme *mycorrhizal roots (Mt/Gv3), and from *M. truncatula *mock-inoculated roots (Mt4) or *M. truncatula*/*G. intraradices *mycorrhizal roots (Mt/Gi4) was used. The *M. truncatula *transgenic plant line p*MtSCP1*::GFP was used and sampling of colonized root pieces was guided by the expression of GFP. Genes selected for analysis were up-regulated ≥2-fold in the Medicago GeneChip^® ^experiment. *TC104979 was reported previously as mycorrhiza-induced. *MtPT4 *was used as positive control and elongation factor 1α (TC106485) was included as endogenous control.

**Figure 5:**
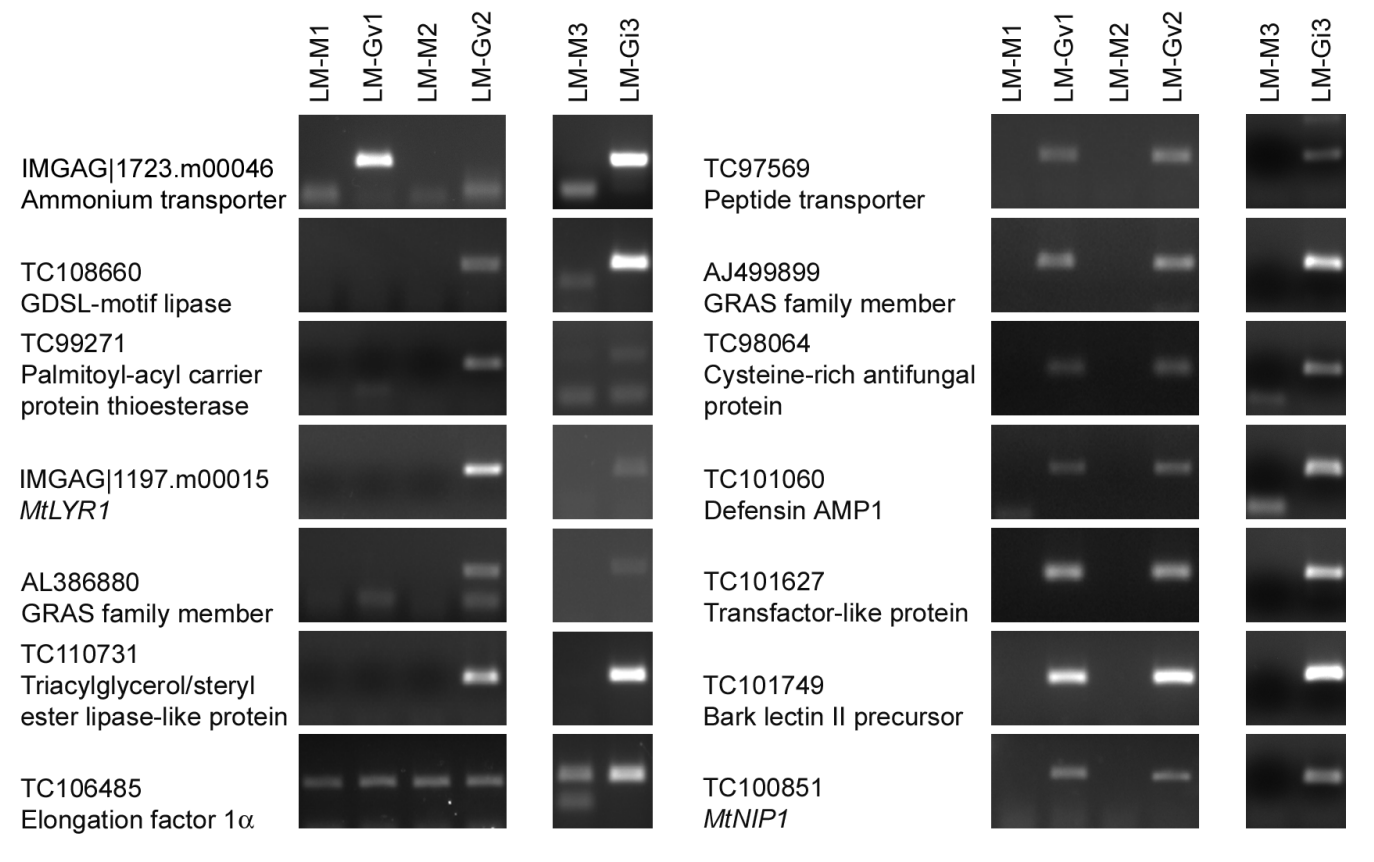
**RT-PCR to detect transcripts of thirteen novel mycorrhiza-induced *M. truncatula *genes in colonized cortical cells**. RNA from cortical cells from three independent mock-inoculated *M. truncatula *root systems (LM-M1, LM-M2 and LM-M3) and cortical cells containing arbuscules from *M. truncatula*/*G. versiforme *mycorrhizal root systems (LM-Gv1 and LM-Gv2) or *M. truncatula*/*G. intraradices *(LM-Gi3) mycorrhizal root systems was used. Cortical cells were obtained by laser microdissection (LM). Transcripts that were induced in only one of the *M. truncatula*/*G. versiforme *LM samples were further evaluated in *M. truncatula*/*G. intraradices *LM samples. The larger size fragment in LM-Gi3 (TC97569) may indicate presence of another peptide transporter gene. TC106485 was used as endogenous control.

Following development and validation of the LM method, we generated cell-type specific RNA samples from cortical cells of *M. truncatula *mock-inoculated roots, and cortical cells containing either *G. versiforme *or *G. intraradices *arbuscules. In addition, we prepared RNA from the highly-colonized 'mycorrhiza-enriched' root pieces from *G. versiforme*-colonized roots. Genes selected for expression analysis in these samples included 6 genes that were previously reported as mycorrhiza-induced, but whose spatial expression patterns were not known, and 12 novel genes that showed increased transcript levels in the GeneChip^® ^experiment (Table 1). Initially, transcript levels were monitored in the RNA from the intact, enriched root piece samples. All 12 new genes showed an increase in transcript levels in the RNA from enriched mycorrhizal root samples relative to that of the mock-inoculated controls confirming the expression patterns observed on the array (Figure 4). This was observed for both *M. truncatula *roots colonized with *G. versiforme *and *G. intraradices*. Transcripts for 9 of these genes were extremely low or not detectable in mock-inoculated roots. In the RNA samples from laser microdissected cells, 13 of 18 genes assayed showed expression in cortical cells containing arbuscules and not in the cortical cells from mock-inoculated control roots (Figure 5). For 6 genes, transcripts were detected in only one of the *G. versiforme *arbuscule-cortical cell samples indicating some variability in the samples. However, analysis in *G. intraradices *arbuscule-cortical cell samples confirmed the expression in this cell-type (Figure 5). It is possible that sample variability arises from variation in the developmental stages of the arbuscules in the sample. This could lead to differences in the samples and would likely have the greatest effects on low abundance transcripts or on genes whose expression is associated with a specific phase of arbuscule development. Using this approach, transcripts of two putative transcription factors of the GRAS family, a peptide transporter, an ammonium transporter, three genes associated with lipid biosynthesis or degradation and a LysM-receptor like kinase were detected in cortical cells containing arbuscules and were not detected in cortical cells from the mock-inoculated controls. Four mycorrhiza-induced genes, the putative copper transport protein, dihydrodipicolinate synthase, inositol polyphosphate phosphatase and an SNF1-like kinase were not detected in cortical cells containing arbuscules. As transcripts of the latter 4 genes were detected in the colonized root pieces (Figure 4), it can be concluded that they are expressed in mycorrhizal roots but in a cell type other than the cortical cells containing arbuscules.

### Analysis of *G. intraradices *gene expression in arbuscules

**Figure 6:**
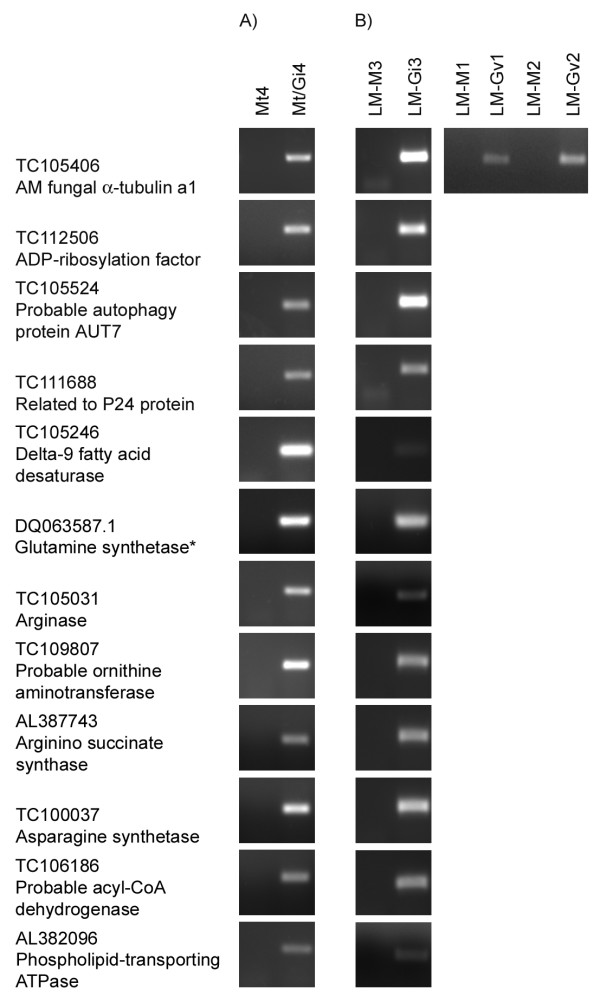
**RT-PCR analysis to detect transcripts of *G. intraradices *genes in mycorrhizal roots and in arbuscules**. Panel A: RNA from whole roots systems of *M. truncatula *mock-inoculated roots (Mt4) and *M. truncatula*/*G. intraradices *mycorrhizal roots (Mt/Gi4). The *M. truncatula *transgenic plant line p*MtSCP1*::GFP was used and sampling of colonized root pieces was guided by the expression of GFP. Panel B: RNA from LM cortical cells from *M. truncatula *mock-inoculated roots (LM-M1, LM-M2 and LM-M3) or cortical cells containing arbuscules from *M. truncatula*/*G. intraradices *mycorrhizal roots (LM-Gi3) or *M. truncatula*/*G. versiforme *mycorrhizal roots (LM-Gv1 and LM-Gv2). Primers designed to TC105406, a putative *G. intraradices *α-tubulin a1 gene amplify α-tubulin from both *G. intraradices *and *G. versiforme*. This was included as positive control in all samples.*G. intraradices *glutamine synthetase was included as an additional positive control.

The expression of 10 *G. intraradices *genes identified on the arrays was evaluated in RNA from both the colonized root pieces and the LM cell-type specific samples (Table 1; Figure 6). The genes selected for analysis included those predicted to encode enzymes of N metabolism, in particular the urea cycle including a putative arginase gene. Consistent with the GeneChip results, transcripts for all 10 genes were present in *M. truncatula*/*G. intraradices *mycorrhizal roots samples. In addition, all genes tested showed expression in arbuscules. This suggests that the urea cycle is likely to be active in this tissue. Previous NMR studies had predicted this but a direct demonstration of the presence of transcripts in arbuscules had not been made. In addition to genes associated with N and amino acid metabolism, we also detected a putative *G. intraradices *AUT7 homolog and a putative phospholipids-transporting ATPase in arbuscules. AUT7 is part of the autophagic machinery and is considered a marker of cells undergoing an autophagic response. The presence of this transcript in arbuscules is the first molecular hint that arbuscule turnover might involve autophagy. The phospholipid-transporting ATPases are P-type ATPases whose activity is required to maintain phospholipid asymmetry in the membrane bilayers [72]. In *Magnaporthe grisea*, the activity of a phospholipid-transporting ATPase was required for development of the penetration hypha and further growth of the fungus within the plant cells [73].

## Discussion

Here, we used the Medicago GeneChip^® ^to extend the transcript dataset for mycorrhizal roots and coupled this with LM and RT-PCR to assess gene expression specifically at the cortical cell-arbuscule interface. At the time that this work was initiated, there were only a few reports of LM of root cells or roots associated with other organisms, consequently, it was necessary to test and modify existing protocols to develop a protocol suitable for *M. truncatula *mycorrhizal roots. The protocol developed was based largely on Kerk et al. [51] and the main modification was the nature of the embedding material. Following initial difficulties with the quality of the RNA extracted from paraffin-embedded tissues, we tested Steedman's wax with the idea that RNA degradation would be minimized by exposing roots to lower temperatures during embedding, and the use of ethanol for de-waxing would be less detrimental than xylene. This proved to be the case and this low-melting-temperature, ethanol-soluble wax enabled us to extract RNA from *M. truncatula *roots of superior quality. LM of approximately 400 *M. truncatula *root cortical cells, followed by two rounds of linear RNA amplification provided microgram quantities of RNA for RT-PCR analyses. These cell-type specific RNA samples were validated by monitoring the expression of three genes, *MtPT4*, *MtSCP1 *and *MtBCP1*, whose expression patterns have been described previously [28,36,38]. Recently, Balestrini et al. [59] reported the use of methacarn fixation and paraffin embedding for LM of cells from tomato roots colonized by the AM fungus *G. mosseae*. However, they did not couple this with linear RNA amplification and consequently, it was necessary to harvest a large quantity of cells, between 3,000 and 11,000 cells to obtain nanogram quantities of RNA. Although the linear RNA amplification used in our approach may lead to a 3' end bias in the transcript molecules, comparative analyses are possible with careful primer design.

The GeneChip analysis provided a greatly expanded view of genes induced in the AM symbiosis, including several transcription factors and receptors. As reported previously, development of the AM symbiosis is accompanied by significant alterations in gene expression including the expression of novel 'mycorrhiza-specific' genes. Surprisingly, transcription factors responsible for mycorrhiza-regulated gene expression are entirely unknown. Here we identified 5 members of the GRAS transcription factor family induced in mycorrhizal roots (AL386879, AL386880, TC105118, IMGAG|1755.m00026, and AJ499899). The GRAS family of proteins is unique to plants and its name derives from three members that were initially identified GAI, RGA, and SCR [74-76]. The Arabidopsis GRAS family contains at least 33 members including Scarecrow and Short Root, two proteins that play a central role in root development (reviewed by [77]. Two *M. truncatula *GRAS proteins, MtNSP1 and MtNSP2, are essential for the activation of Nod-factor-induced genes in the rhizobial symbiosis [62,63]. Given their roles in root development and symbiosis, members of this family are likely candidates for regulating gene expression in mycorrhizal roots. Here, we confirmed by RT-PCR that two putative GRAS genes; AJ499899 and AL386880, were expressed in *G. versiforme*- or *G. intraradices*-colonized roots, with non-detectable or low levels of transcripts in mock-inoculated roots. Furthermore, AJ499899 and AL386880 transcripts were not detected in cortical cells from mock-inoculated roots but were detected in arbuscule-containing cortical cells. Consequently, it is possible that these GRAS factors regulate gene expression in colonized cortical cells. The current data do not preclude a role in other cell types and one GRAS factor, represented by EST AL386880 is also highly expressed in root nodules. It would be interesting to know whether expression is associated with cells containing symbiosomes [60].

In addition to transcription factors, receptor proteins operating in signaling pathways, involved in arbuscule development are also entirely unknown. In contrast, receptors involved in signaling during nodule development have been described. Genes predicted to encode LysM-RLK including NFR1 and NFR5 in *Lotus japonicus *[78,79] and orthologs LYK3 and NFP in *M. truncatula *[80], and SYM10 in pea [78] are involved in Nod factor signaling and are likely to be Nod factor receptors. The *M. truncatula *LysM-RLK family contains at least 17 genes, which, based on their kinase domain phylogeny, are divided into three subfamilies. Subfamily II contains NFP and four other members of the family of which MtLYR1 is most similar in sequence to NFP. There are no ESTs for *MtLYR1 *and in previous studies, transcripts were detected in nodulated roots but not in nodules [65]. Here, we found that *MtLYR1 *(IMGAG|1197.m00015) transcripts increased in *G. versiforme *or *G. intraradices*-colonized roots. Moreover, the *MtLYR1 *gene was detected in cortical cells with arbuscules. Like NFP, MtLYR1 has three LysM domains and a Ser-Thr kinase domain that lacks the activation and P-loops and consequently, it probably lacks kinase activity [65]. It is possible that these 'inactive' kinases dimerize with an active receptor kinase to form a receptor with kinase activity and consistent with this, heterodimerization of NFR1 and NFR5 has been demonstrated. LysM domains recognize N-acetyl glucosamine or structurally similar molecules such as Nod factors [81-83]. It is possible that the LysM domain of MtLYR1 recognizes the N-acetyl glucosamine residues in chitin in the AM fungal cell walls and given its expression pattern, it could play a role in signaling during arbuscule development.

Cell biological analyses of the arbuscule-cortical cell interface suggest that the peri-arbuscular membrane increases the membrane area within the cortical cell at least 10-fold. Consistent with this, metabolite profiles of mycorrhizal roots indicate a significant increase in fatty acids [84]. The GeneChip analyses identified two new mycorrhiza-induced genes predicted to be involved in fatty acid metabolism; a putative palmitoyl-ACP thioesterase gene (TC99271) and a triacylglycerol/steryl ester lipase-like protein gene (TC110731) that show a 49- and 102-fold increase in transcript levels respectively. RT-PCR analysis indicated that TC99271 and TC110731 transcript levels increase in cortical cells with arbuscules. Fatty acid synthesis in plants is a cyclic process and the extension cycles involves the binding of acyl chains to a soluble acyl carrier protein (ACP), and specific thioesterases hydrolyze the newly formed acyl-ACP into fatty acids and ACP [85]. An increase in palmitoyl-ACP thioesterase gene expression suggests an increase in the biosynthesis of palmitic acid. Consistent with this, a recent metabolite profile of *G. intraradices*/*M. truncatula *roots showed that mycorrhizal roots contained high levels of palmitic and oleic acids during active phases of AM symbiosis [84]. Oleic acid (18:1Δ^9^) is created by a desaturation reaction catalyzed by a delta-9 desaturase. Surprisingly, a *G. intraradices *delta-9 desaturase gene was expressed highly in mycorrhizal roots and specifically in cells with arbuscules. Previous studies had speculated that the fungus might obtain palmitic acid from the plant, and even suggested that AM fungi might lack fatty acid synthase activity and thus be dependent on host lipid [86]. In the absence of a full genome sequence, it is not possible to determine whether this is correct, however, our findings of complementary gene expression patterns in the two symbionts is intriguing.

Other metabolic changes documented in mycorrhizal roots include an increase in amino acids [84]. In plants, there are at least three protein families involved in transport of small peptides, the ABC transporters, the di/tripeptide transporters (PTR family) and the oligopeptide (OPT) family. Two genes (AL387494 and TC97569) of the PTR family were expressed in mycorrhizal roots and TC97569 was detected in cells containing arbuscules. The GeneChip experiment also led to the identification of other new genes predicted to encode transport proteins including a copper transport protein (TC97522) and an ammonium transporter (IMGAG|1723.m00046) and the latter was expressed in cells with arbuscules. The identification of a mycorrhiza-induced ammonium transporter is particularly interesting. It is clear from many studies that in addition to Pi, N is also transferred from the fungus to the plant and recent analyses indicate that N is likely transferred to the plant as ammonium. This ammonium transporter is a strong candidate for a role in the acquisition of ammonium delivered via the fungus. Metabolic labeling studies and gene expression analyses led to a model in which inorganic N taken up by the fungal extra-radical mycelium is converted into arginine which is transported into the intra-radical mycelium, where it is broken down into amino acids. Ammonium, resulting from arginine breakdown is proposed to be exported to the plant [29]. In further support of the labeling studies, Govindarajulu et al. [29] showed elevated transcripts of *G. intraradices *ornithine aminotransferase, a urease accessory protein and an ammonium transporter in the intra-radical mycelium relative to the extra-radical mycelium.

Interestingly, the *G. intraradices *genes present on the *M. truncatula *array include genes predicted to encode enzymes of the urea cycle as well as amino acid metabolism. RT-PCR analysis showed that these genes were expressed in *G. intraradices*-mycorrhizal roots, and analysis of the cell-type specific samples, indicates that they are expressed in the arbuscules. These data extend the previous findings, showing that the genes of urea and amino acid metabolism are expressed in the arbuscules. In addition, our data set includes a gene predicted to encode arginase that breaks down arginine to release ornithine and urea. This is a key step for the release of ammonia and further supports the model proposed by Govindarajulu et al. [29]. In addition to the urea cycle, we detected *G. intraradices *asparagine synthetase and glutamine synthetase gene expression in arbuscules. This suggests that ornithine, released via arginase activity, may be cycled into asparagine and glutamine through the action of asparagine synthetase and glutamine synthetase. Interestingly, the metabolite profiling studies found higher levels of specific amino acids, including Glu, Asp, and Asn in mycorrhizal roots [84]. From these studies, it is not possible to determine whether the amino acids are in the plant or AM fungus, however, coupled with the gene expression studies, there is evidence to suggest that at least some of this amino acid accumulation may result from activities in the fungus.

## Conclusion

Using a combination of arrays, LM and RT-PCR, we have extended the *M. truncatula/G. intraradices *mycorrhizal root transcriptome and identified novel *M. truncatula *and *G. intraradices *genes that are expressed in cortical cells harboring arbuscules. By virtue of their expression patterns, the genes expressed in this cell type are candidates for mediating aspects of development or function of the symbiotic arbuscule-cortical cell interface. Novel genes potentially involved in signaling and in N transport and metabolism are of particular interest. This dataset will be valuable for future studies aimed at determining the molecular events that control arbuscule development, and N transport and metabolism in the AM symbiosis.

## Methods

### Plant transformation

The *pMtSCP1*::GFP construct described in Liu et al. [36] was introduced into *Agrobacterium tumefaciens *(LBA4404) and was used to transform *Medicago truncatula *cv. Jemalong, (A17) according to Trieu and Harrison [87] with minor modifications as described in Javot and Harrison [88]. Regenerated plants were selected with 1 mg L^-1 ^bialaphos and were allowed to self pollinate. The progeny were tested to verify the presence of the *GFP *and *Bar *genes, and were inoculated with *G. versiforme *to verify the mycorrhiza-inducible expression of sGFP [36]. Seed from one transgenic line MtSCP-GFP1-1 (from the T3 or T4 generations) were used in the LM experiments.

### Plant material, growth conditions and inoculation methods

Plants were grown in a growth room under a 16 h light (25°C) and 8 h dark (22°C) period. *G. intraradices *was maintained in cultured carrot roots on plates, and spores were prepared from the plates as described [89]. Stock cultures of *G. versiforme *was maintained on leek plants or Bahia grass in pot culture and the preparation of surface-sterilized spores has been described previously [36,90,91]. Plants were supplied with half-strength Hoagland's solution containing 20 μM potassium phosphate twice a week.

For the GeneChip experiment, the root tissue was from the same experiment as that described in Liu et al. [39]. Briefly, 14 day old *M. truncatula *(A17) plants were inoculated with 8,000 *G. intraradices *spores/pot and grown for 30 days. Roots were harvested and frozen in liquid nitrogen. Random samples were collected for the assessment of their colonization levels with the gridline intersect method [92]. The colonization levels of the three *G. intraradices *inoculated replicates used in the array experiment were 49, 50 and 53% root length colonized (RLC) as reported in Liu et al. [39]. For all other experiments, the *M. truncatula *transgenic line *pMtSCP1*::GFP (T_3 _or T_4 _generation) was used.

To obtain highly colonized root pieces, 26 day old *pMtSCP1*::GFP *M. truncatula *plants were transplanted in large cones (4 × 20 cm: 1 plant per cone) and were inoculated with 800 *G. versiforme *spores per plant or mock-inoculated with the last rinse from the spore surface sterilization procedure as described previously [36,90,91]. At 28 days post-inoculation, roots were examined under a stereomicroscope equipped with UV-light, and regions of the root system that showed green fluorescence (Additional file 3) were dissected out and immediately frozen in liquid nitrogen, and stored at -80°C for RNA isolation. Likewise, mock-inoculated pieces of roots were harvested in a similar manner from similar regions of the mock-inoculated root systems.

For the LM experiments, 15 day old or 23 day old *pMtSCP1*::GFP *M. truncatula *plants were transplanted in large cones and inoculated with 1000 *G. versiforme *spores per plant or 1000 *G. intraradices *spores per plant, or mock-inoculated as described previously. Plants were harvested at 22 or 26 days post-inoculation with *G. versiforme *or *G. intraradices*, respectively. The harvest of root sections took place during the evening hours to facilitate the tissue preparation for LM.

### Tissue preparation and laser microdissection

The method described was based on that of Kerk et al. [51] with modifications. Instead of paraffin wax, the low melting temperature Steedman's wax was used for embedding root tissues. Steedman's wax was prepared by melting 900 g polyethylene glycol 400 distearate (Sigma-Aldrich, St. Louis, MO) and 100 g 1-hexadecanol (Sigma-Aldrich) at 65°C, and it was mixed thoroughly with a stirring bar. The wax was stored in 50-ml sterile tubes and only the volume needed was melted at 38°C.

*M. truncatula SCP1*::GFP root systems were submerged in DEPC-treated autoclaved water and colonized regions were selected based on expression of GFP as described above. Mock-inoculated root pieces were collected in a similar manner. Root pieces of between 4 and 8 mm long were dissected and immediately transferred into 20-ml RNase-free scintillation vials containing freshly prepared Farmer's fixative (3:1 (v/v), ethanol: acetic acid). Roots were incubated in the fixative for 12 h with rotation at 4°C, followed by dehydration also at 4°C, in the following ethanol series: 75% (v/v), 85%, 95% (with 0.1% eosin y), 100% and 100% with transfer every 2 h. The quality of RNA extracted from tissues in one block was evaluated by the bioanalyzer (RIN = 6.7).

After the last ethanol step, half of the ethanol was removed and the vials were incubated at 38°C for 10 min prior to adding the molten Steedman's wax. Vials with 1:1 ethanol: wax, were incubated for 12 h at 38°C, and followed by 3 changes of 100% wax at 2 h intervals. Roots were embedded in paper boats at room temperature. Blocks were stored in plastic bags until they were processed.

Sectioning with Steedman's wax was performed at cool room temperature (less than 23°C). Transverse root sections of 10–15 μm were made using a Leica RM 2235 rotary microtome (Leica Microsystems Inc., Bannockburn, IL). Ribbons were arranged on UV-treated, 1 mm PEN-membrane covered slides (P.A.L.M. Microlaser Technologies, Bernried, Germany) and they were stretched with a few drops of DEPC-treated autoclaved water. Slides were kept in a slide warmer at 32°C overnight. Sections were de-waxed by pipetting 100% ethanol with a transfer pipette several times until the wax was not visible anymore, and then dried at 32°C before the cell harvest.

The P.A.L.M. Microbeam system with a Robomover (P.A.L.M. Microlaser Technologies) was used for LM. Cortical cells that contained arbuscules or cortical cells from mock-inoculated roots were counted and marked (Additional files 3, 6). The contour of a cell or group of cells was cut twice with the laser and target cells were automatically catapulted into the cap of a microcentrifuge tube that contained RLT buffer (Qiagen Inc., Valencia, CA) with β-mercaptoethanol. We harvested cortical cells containing *G. versiforme *arbuscules or mock-inoculated controls from 2 biological replicates (each plant was a replicate). For *G. intraradices*, cells from 3 plants were pooled as shown in the Additional file 4. Cells were stored at -80°C until RNA isolation.

### RNA isolation

For the GeneChip Medicago genome arrays, root tissue from 3 biological replicates (*G. intraradices*- or mock-inoculated plants) were ground in liquid nitrogen using a mortar and pestle. Total RNA was extracted with Trizol reagent (Invitrogen Corporation, Carlsbad, CA) with additional phenol (pH 6.6): chloroform (1:1, v/v) and chloroform purification steps. RNA was treated with Turbo DNase I (Ambion Inc., Austin, TX), and column purified with an RNeasy MinElute CleanUp kit (Qiagen). RNA was quantified using a Nanodrop Spectrophotometer ND-100 (NanoDrop Technologies, Willington, DE) and evaluated for purity with the bioanalyzer.

For the small dissected root samples, root pieces harvested from 3 plants (*G. versiforme*- or mock-inoculated) were pooled and ground with a disposable pellet pestle in a centrifuge tube containing liquid nitrogen. Total RNA from root sections or LM cortical cells was isolated with the RNeasy Micro kit (Qiagen) according to the manufacturer's protocol.

### RNA amplification

Five hundred nanograms of total RNA isolated from the dissected root pieces were amplified using the TargetAmp 1-round aRNA amplification kit (Epicentre Biotechnologies, Madison, WI). 3 μg of aRNA or total RNA [*G. versiforme*- or mock-inoculated aRNA, and *G. intraradices*- or mock-inoculated RNA] were then treated twice with 1 Unit of Turbo DNase (Ambion Inc., Austin, TX). After an incubation of 20 min at 37°C each time, samples were purified using phenol (pH6.6): chloroform (1:1, v/v) and chloroform, followed by isopropanol precipitation.

Total RNA from LM cortical cells was amplified using the TargetAmp 2-round aRNA amplification kit (Epicentre Biotechnologies) and the aRNA yields are listed in Additional file 4.

### Oligonucleotide design

Primers were designed using Primer3 software [93] and the product sizes were less than 180 bp. To avoid non-specific product formation, oligonucleotides were designed with reduced self and cross dimer ΔG using NetPrimer software (PREMIER Biosoft International, Palo Alto, CA). Most primers were designed near the 3' end of the coding sequences or 3' UTR. All the primers that were used are listed in Additional file 7. Primers designed to detect a *G. versiforme *ferritin-like gene were based on the sequence in Additional file 8.

### cDNA synthesis and PCR conditions

First-strand cDNA was synthesized from 1.5 μg of DNase-treated total RNA using SuperScript III (Invitrogen) with 500 ng anchored oligo (dT) primers in a 20 μl reaction. For the aRNA, 600 ng of DNase-treated aRNA (1-round) was reverse-transcribed using 500 ng random hexamers in a 40 μl reaction. This reaction was incubated at room temperature for 5 min prior to 2 h cDNA synthesis at 50°C and 15 min at 70°C. Following cDNA synthesis, either 110 μl or 35 μl of water were added to cDNA samples derived from 600 ng aRNA or 1.5 μg total RNA, respectively. Each PCR reaction consisted of 1 μl cDNA in a 20 μl reaction with the following components: 4 μl 5× GoTaq green reaction buffer, 0.4 μl 10 mM dNTP mix, 0.4 μl each primer (10 μM), 0.5 Units of GoTaq DNA polymerase (Promega Corporation, Madison, WI). The thermal profile consisted of incubation at 94°C for 1 min, followed by 30 cycles at 94°C for 45 sec, maximum annealing temperature (*see *Additional file 7) for 45 sec, 72°C for 45 sec, and a final incubation at 72°C for 5 min.

For RT-PCR of the LM aRNA samples, the cDNA was generated using gene-specific primers. Seventy five ng of aRNA was mixed with 1 μl 10 mM dNTP mix and 0.25 μl of gene-specific forward primer (10 μM), and incubated at 65°C for 5 min. 4 μl of 5× first-strand buffer, 1 μl 0.1 M DTT, 10 Units of RNase OUT, 5 Units of SuperScript III reverse transcriptase and DEPC-treated autoclaved water up to 20 μl were added. cDNA synthesis was performed at 50°C for 2 h, followed by inactivation at 70°C for 15 min. Each 20 μl PCR reaction included 5 μl of cDNA, 2 μl 10× PCR buffer, 0.4 μl 10 mM dNTP mix, 0.4 μl each primer (10 μM), 0.5 Units of HotStarTaq DNA polymerase (Qiagen) and autoclaved water. In order to minimize non-specific priming during amplification, we used touchdown PCR as described previously [94]. The annealing step was carried out at 5°C above and 3°C below the primer's melting temperature as shown in the Additional file 7. The touchdown thermal profile consisted of a 15 min heat activation step at 95°C, followed by 10 cycles at 94°C for 30 sec, a ramp of -0.8°C per cycle (*see *Additional file 7 for max and min temperatures) for 30 sec, 72°C for 1 min. Additional 38 cycles at 94°C for 30 sec, minimum annealing temperature (*see *Additional file 7) for 30 sec, 72°C for 1 min, and a final incubation at 72°C for 10 min. All the PCR products were visualized using 2% TAE-agarose gels stained with ethidium bromide. The PCR product of one LM sample (TC110731) was sequenced to confirm identity.

### GeneChip^® ^Medicago genome array labeling and hybridization

The Affymetrix GeneChip^® ^Medicago Genome Array (Affymetrix, Santa Clara, CA) was used for expression analysis. RNA from three independent biological replicates was analyzed for each treatment. 5 μg of column-purified total RNA were used for one-cycle eukaryotic target labeling. Targets were hybridized to the arrays; probe arrays were washed, stained and scanned according to the manufacturer's instructions (Affymetrix).

### GeneChip^® ^data acquisition and analysis

Data normalization between chips was conducted using Robust Multi-chip Average (RMA) [95]. Presence/absence call for each probe set was obtained using dCHIP [96]. Gene selections based on associative *t*-test [97] were made using MATLAB^® ^(MathWorks, Natick, MA). Details of this method were presented previously [60]. A selection threshold of 2 for transcript ratios (where applicable) and a Bonferroni-correction *p*-value threshold of 1.14 × 10^-6 ^were used. False discovery rate of all significant genes was monitored with q-values obtained by EDGE software [98,99].

## Authors' contributions

SKG developed the laser microdissection method, carried out the laser microdissection and the RT-PCR analyses, and wrote the manuscript. HJ and PD generated the *M. truncatula *transgenic plant line used for the laser microdissection studies and HJ reviewed the manuscript. ITJ carried out the affymetrix chip experiments and YT analyzed the affymetrix array data. EBB developed the laser microdissection method and reviewed the manuscript. MKU participated in the affymetrix array experiments and reviewed the manuscript. MJH conceived the experiments and wrote the manuscript. All authors read and approved the final manuscript.

## Supplementary Material

Additional file 1 **Genes showing differential expression in *Medicago truncatula *roots colonized by *Glomus intraradices *relative to mock-inoculated control roots.** Click here for file

Additional file 2 **Probesets on the GeneChip^® ^predicted to represent *Glomus intraradices *genes.** Click here for file

Additional file 3 **M. truncatula pMtSCP1::GFP plant line used for laser microdissection.** (a) *M. truncatula pMtSCP1::GFP *roots colonized by *G. intraradices*. (b) Mock-inoculated *M. truncatula pMtSCP1::GFP *roots. (c and e) Transverse sections of *M. truncatula pMtSCP1::GFP *mock-inoculated roots and (d and f) roots colonized by *G. intraradices*. Click here for file

Additional file 4 **Number of *Medicago truncatula *cortical cells obtained by laser microdissection and laser pressure catapulting, and aRNA yield after two rounds of linear RNA amplification.** Click here for file

Additional file 5 **Analysis of the *MtPT4* transcripts by RT-PCR.** (A) RNA from LM cortical cells from mock-inoculated roots (LM-M) and *M. truncatula/G. versiforme *mycorrhizal roots (LM-GV). Glyceraldehyde 3-phosphate dehydrogenase (GDPD) was used as endogenous control. *M. truncatula*/*G. versiforme *whole mycorrhizal root system (MtGV1) and *M. truncatula*/*G. versiforme root pieces *(MtGV2) samples were included as positive controls. (B) Location of oligonucleotide primers based on the ATG site of the *MtPT4 *(AY116210) coding sequence, 74–280 bp (*MtPT4*-a), 385–586 bp (*MtPT4*-b), 468–677 bp (*MtPT4*-c), and 1438–1545 bp (*MtPT4*-d). Click here for file

Additional file 6 **Laser microdissection of cortical cells from *M. truncatula* roots**. *pMtSCP1::GFP* mock-inoculated (1A-1D) roots or *M. truncatulapMtSCP1::GFP/G*. *versiforme* mycorrhizal roots (2A-2D) were used for LM. Transverse sections of mock-inoculated roots (1A) and mycorrhizal roots (2A) with outlined target cortical cells, path of laser ablation (1B, 2B), target areas for laser pressure catapulting (1C, 2C), and view after cell catapulting (1D, 2D). Bar = 25 μm. Click here for file

Additional file 7 **Oligonucleotides used for RT-PCR.** Click here for file

Additional file 8 **Sequence of TC86704 predicted to encode a *G. versiforme* ferritin-like protein.** This TC is assembled from ESTs from a *Glomus versiforme *spore cDNA library. Several ESTs were sequenced and they assembled into TC85704 that represents almost the complete coding sequence of the gene. The predicted protein shares over 54% amino acid identity with ferritin from *Triatoma infestans*, a blood sucking arthropod and 52% identity with ferritin from *Suberites domuncula*, a marine sponge. The finding of a putative *G. versiforme *ferritin-like gene is surprising because based on BLAST searches, sequences similar to this are not present in other fungi. In general fungi use diverse approaches to obtain and store iron including a range of siderophores such as ferricrocin or hydroxamate-type siderophores that are synthesize by non-ribosomal peptide synthetases [100-102]. Click here for file
