# Supplementary figures and images for: Medicago truncatula and Glomus intraradices gene expression in cortical cells harboring arbuscules in the arbuscular mycorrhizal symbiosis
Source: BMC Plant Biol. 2009 Jan 22;9:10. doi: 10.1186/1471-2229-9-10 (PMC2649119; doi:10.1186/1471-2229-9-10)

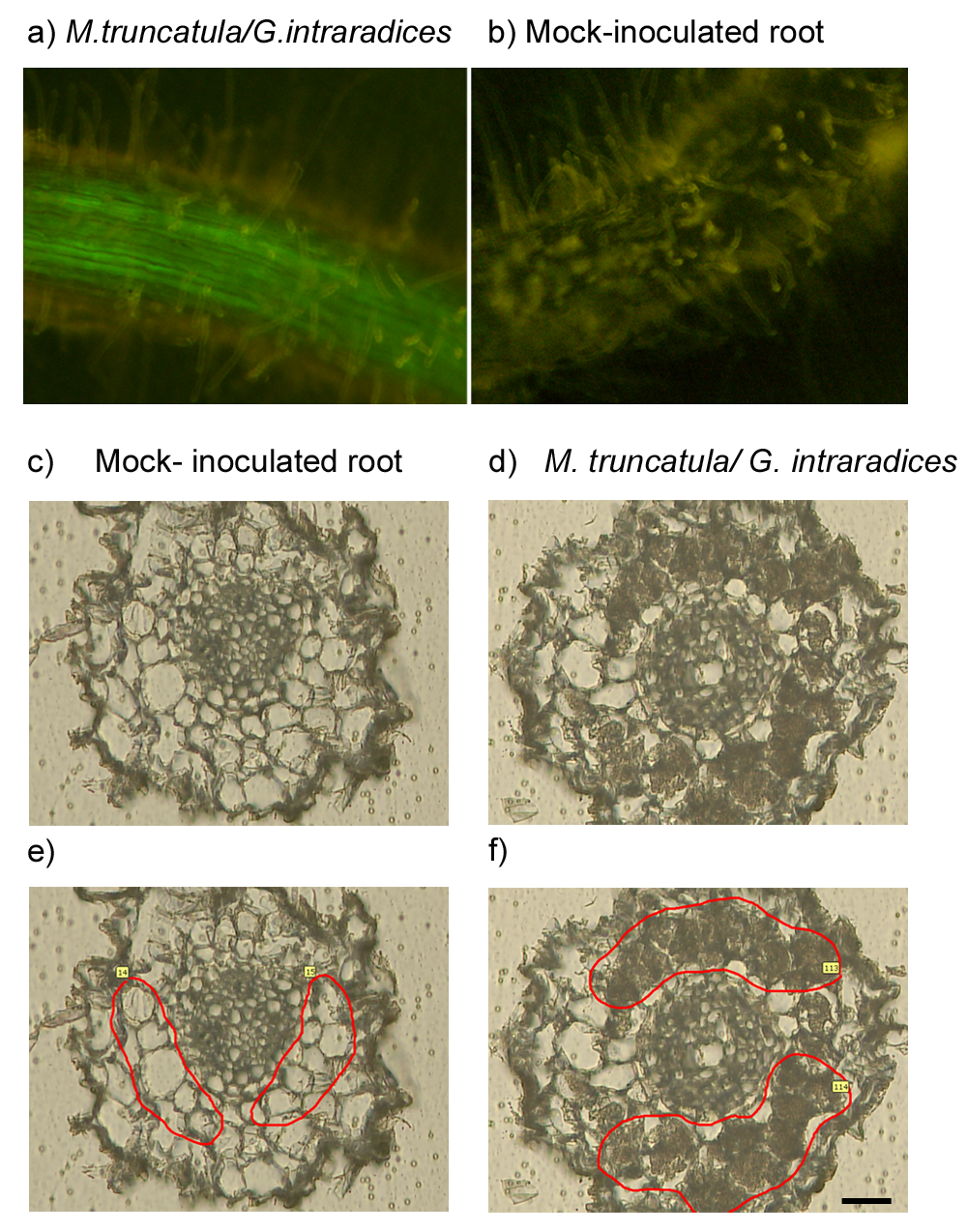

Supplement: Additional file 3 — M. truncatula pMtSCP1::GFP plant line used for laser microdissection. (a) M. truncatula pMtSCP1::GFP roots colonized by G. intraradices. (b) Mock-inoculated M. truncatula pMtSCP1::GFP roots. (c and e) Transverse sections of M. truncatula pMtSCP1::GFP mock-inoculated roots and (d and f) roots colonized by G. intraradices. [file 1471-2229-9-10-S3.tiff]

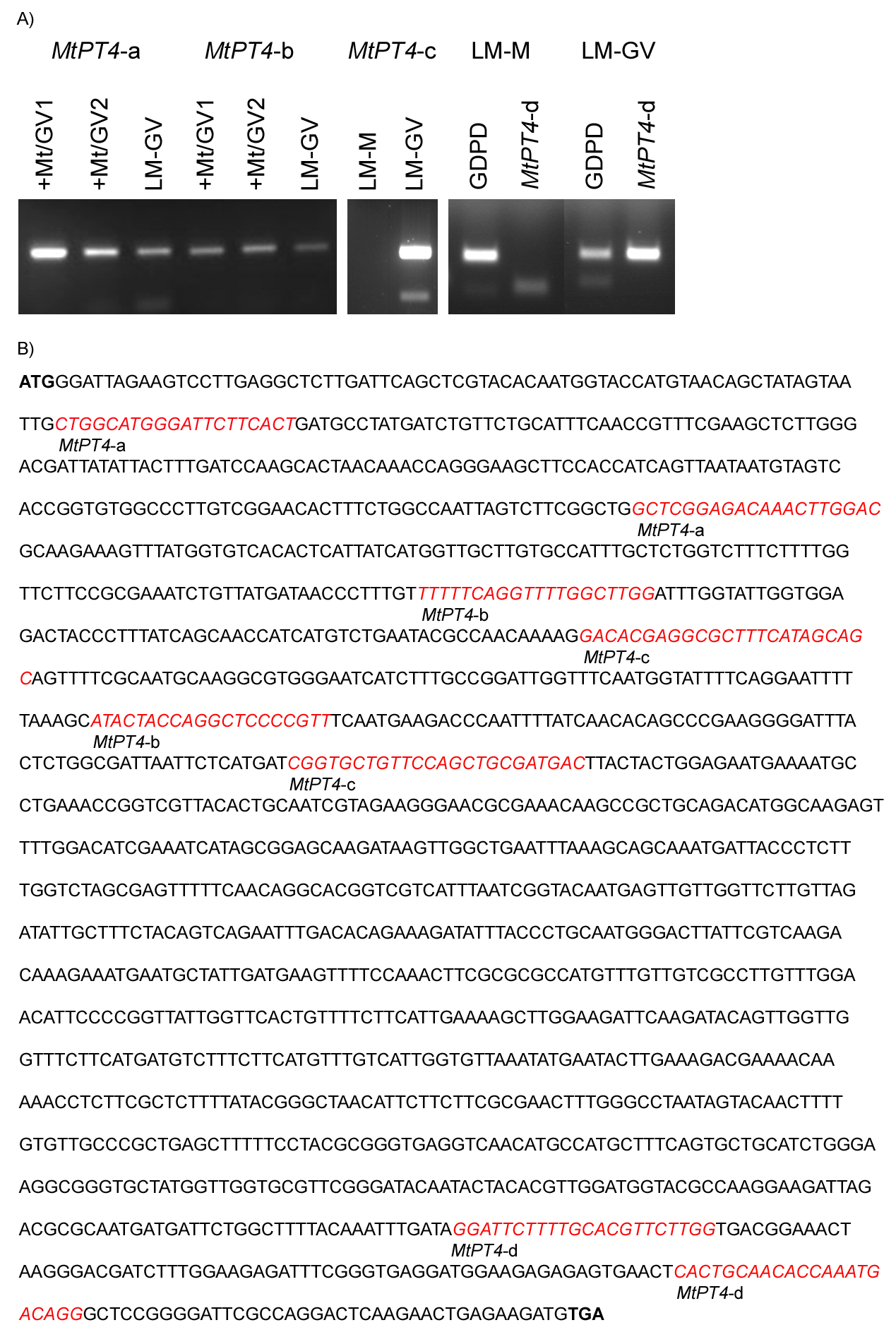

Supplement: Additional file 5 — Analysis of the MtPT4 transcripts by RT-PCR. (A) RNA from LM cortical cells from mock-inoculated roots (LM-M) and M. truncatula/G. versiforme mycorrhizal roots (LM-GV). Glyceraldehyde 3-phosphate dehydrogenase (GDPD) was used as endogenous control. M. truncatula/G. versiforme whole mycorrhizal root system (MtGV1) and M. truncatula/G. versiforme root pieces (MtGV2) samples were included as positive controls. (B) Location of oligonucleotide primers based on the ATG site of the MtPT4 (AY116210) coding sequence, 74–280 bp (MtPT4-a), 385–586 bp (MtPT4-b), 468–677 bp (MtPT4-c), and 1438–1545 bp (MtPT4-d). [file 1471-2229-9-10-S5.tiff]

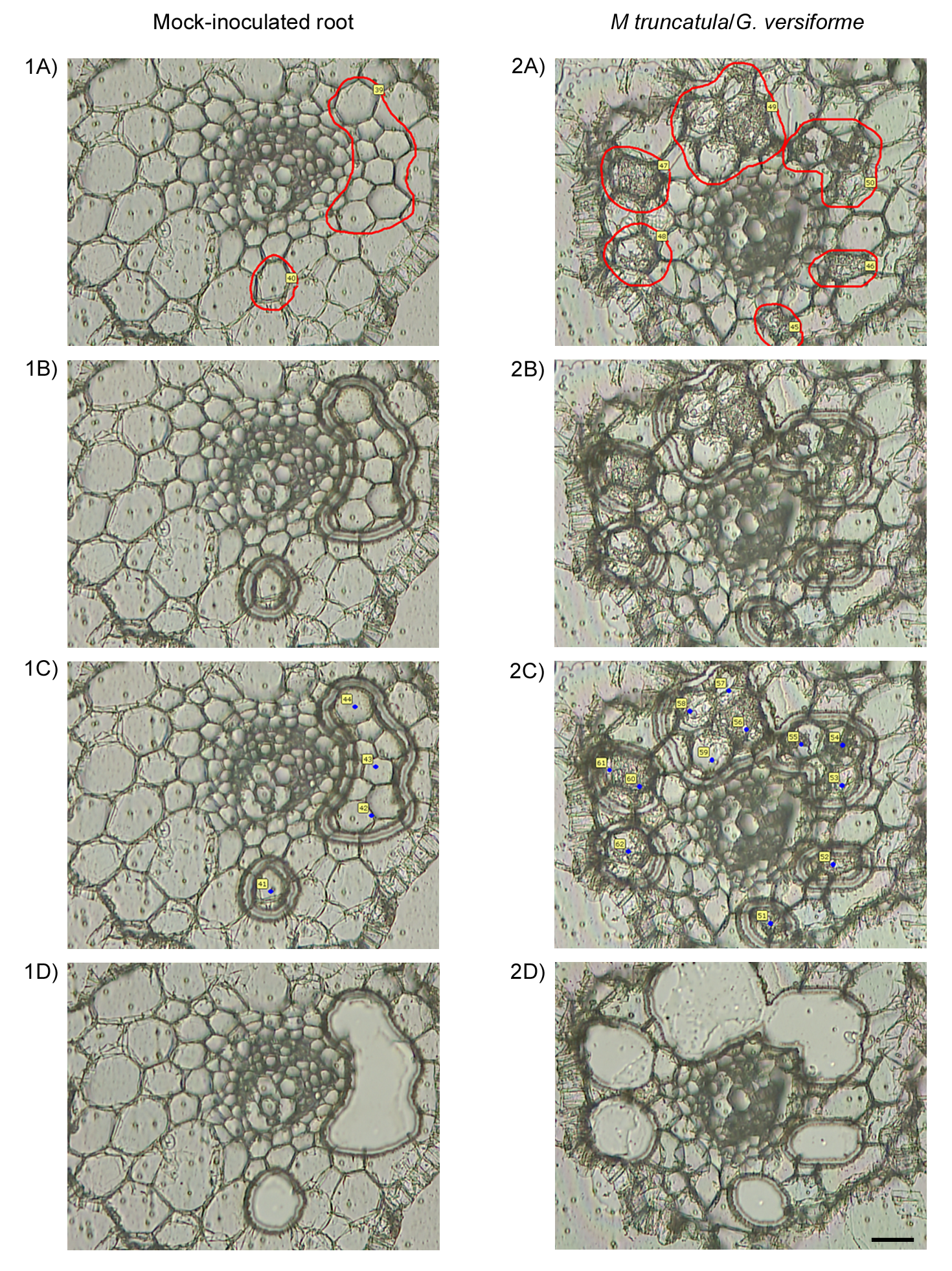

Supplement: Additional file 6 — Laser microdissection of cortical cells from M. truncatula roots. pMtSCP1::GFP mock-inoculated (1A-1D) roots or M. truncatulapMtSCP1::GFP/G. versiforme mycorrhizal roots (2A-2D) were used for LM. Transverse sections of mock-inoculated roots (1A) and mycorrhizal roots (2A) with outlined target cortical cells, path of laser ablation (1B, 2B), target areas for laser pressure catapulting (1C, 2C), and view after cell catapulting (1D, 2D). Bar = 25 μm. [file 1471-2229-9-10-S6.tiff]
