# Supplementary material for: Medicago truncatula and Glomus intraradices gene expression in cortical cells harboring arbuscules in the arbuscular mycorrhizal symbiosis
Source: BMC Plant Biol. 2009 Jan 22;9:10. doi: 10.1186/1471-2229-9-10 (PMC2649119; doi:10.1186/1471-2229-9-10)
Supplement: Additional file 8 — Sequence of TC86704 predicted to encode a G. versiforme ferritin-like protein. This TC is assembled from ESTs from a Glomus versiforme spore cDNA library. Several ESTs were sequenced and they assembled into TC85704 that represents almost the complete coding sequence of the gene. The predicted protein shares over 54% amino acid identity with ferritin from Triatoma infestans, a blood sucking arthropod and 52% identity with ferritin from Suberites domuncula, a marine sponge. The finding of a putative G. versiforme ferritin-like gene is surprising because based on BLAST searches, sequences similar to this are not present in other fungi. In general fungi use diverse approaches to obtain and store iron including a range of siderophores such as ferricrocin or hydroxamate-type siderophores that are synthesize by non-ribosomal peptide synthetases [100-102]. [file 1471-2229-9-10-S8.doc]

**Additional file 8**

*G. versiforme* ferritin heavy chain-like sequence (TC86704)

GATATAAAGACCAACGATTCTTTGGCTTTTCAGAACAATTAATTCTTTACAAACAAATGAGCAAAATGAATCAATCCAGTGCTAAAACTACTTCTTACTCTAATGATCTTCAAAAAGCAATCAATGGTCAAATTAATAACGAAATCTGTGCCAGTCATGAGTATTTCCAGCTAGCTTGGTCTTGCGCTAGGGATGAAAATGCTCTTCATGGATTTAAAGAATTTTTCTTGAACTTCGCTCACCAACGTTTTTGCGATGCAATGTGTTTATCAAGTTATCAAACTGTACGTGGTGGTCACGTTGAAGTTTCGGAAACCAGGGCACCAAAGTTGAACTGGAAAGGAGCTGAAGAAACCTTTAAAAAGCTTTTGGAACTTGAAAAGGGTATCTCTGATGATTTGAACAAACTCGACGATACCGCTGATAAAACGGGTGATAGAGCAGCTGCAAACTTTATCGAAAACAAATTGTTGGCAAAACAAACTTGTCGTGTCAAGATTATAGCGGACATGCTTACACAAATTAAACGTGTTAGTGGAGAAGGTACCGGTTTGTTCCAACTTGATTTGGTATTGAAAAAAAATTGTGGAATTCCTCCTTGGGATGAAAGACACGATGATTACAGACGCGAACACGGACACGGACGCGAACACGAACGCGAACACGAACGTATTGCTGCCTTTCACGAACGTTTTGCTGCTAATTTGAACCTTAAAAATTGAAAAAAATCACGTTCAGCTTAATTATTTTTTATTGCTGATTTAGTTTAAAGAAGCTTCATGTAATAATTAATCCTTTTGTAAATTGGATATTTGTTAATAATTTTTCCAATAAAATTTATTTTTTTTTTGATGTAAAA
